# SUNRED, a natural extract-based biostimulant, application stimulates anthocyanin production in the skins of grapes

**DOI:** 10.1038/s41598-019-39455-0

**Published:** 2019-02-22

**Authors:** Qunxian Deng, Hui Xia, Lijin Lin, Jin Wang, Lu Yuan, Kangning Li, Jinrong Zhang, Xiulan Lv, Dong Liang

**Affiliations:** 1 College of Horticulture, Sichuan Agricultural University, Chengdu, Sichuan 611130 China; 2 Chengdu Agricultural College, Chengdu, Sichuan 611130 China; 3 Sichuan Academy of Botanical Engineering, Neijiang, Sichuan 641200 China

## Abstract

Anthocyanins are important components in skins of red table grapes and contribute to the berries appearance, a key quality characteristic for customers. In recent years, exogenous foliage fertilizers has been applied to grapevines to improve the pigmentation of the fruit. The present study examines the effect on a biostimulant (SUNRED) pre-*véraison* application in the accumulation of anthocyanins in ‘Red Globe’ grapes, and investigates the related changes in expression of key genes and their enzyme activities in the flavonoid pathways. Additionally, abscisic acid (S-ABA) was also applied to grapevines to evaluate the comparative effect of SUNRED. Our analyses showed that total anthocyanin contents increased in both SUNRED and S-ABA treated grapes; for S-ABA, a 1% dilution (A100) of the commercially available stock solution treatments represented the greatest effect on pigmentation; for SUNRED, a 0.1% dilution (S1000) was most effective. The anthocyanin contents increased by 1.16-fold and 1.4-fold after A100 and S1000 treatments, respectively. The gene expression analyses showed that almost all genes involved in the anthocyanin biosynthesis pathway up-regulated after A100 and S1000 treatments, suggesting that the increment in total anthocyanin content was attributed to the increased expression level of related genes. Moreover, the activities of phenylalanine ammonia-lyase (PAL), chalcone isomerase (CHI), UDP glucose: flavonoid 3-o-glucosyl transferase (UFGT) and dihydroflavonol 4-reductase (DFR), key enzymes for biosynthesis of anthocyanin, were increased by the exogenous treatments. Overall, our findings clearly demonstrate that application of exogenous biostimulant have a positive effect on the pigment characteristics of grape crop.

## Introduction

Grape (*Vitis vinifera* L.) is one of the most extensively cultivated fruit crops around the world. Between 2005 and 2015, the cultivation area and production of grapes in China increased by more than 1.96-fold (408,000 to 799,000 hm^2^) and 2.36-fold (5,794,000 to 13,669,000 tones) respectively. As a consequence, China has become the largest producer of table grape since 2011 (data from the Ministry of Agriculture of China).

In the last decade, cultivation of table grapes in China has spread from the eastern and northern regions to the western and southern regions. Indeed, some southern provinces, such as Jiangsu, Guangxi, Yunnan and Sichuan, have become the important areas of grape industry in China. However, vineyards in southern China may be affected by low light conditions, a high rainfall, and relatively low thermal amplitude during *véraison* to maturation, which adversely affect fruit maturation indexes, such as color development and biosynthesis of primary or secondary metabolites^1^. To overcome these problems, exogenous hormones can be applied to red-colored table grapes to improve their internal and external appearances and quality, for instance, abscisic acid^2^, methyl jasmonate^3^, brassinosteroids^4^, and 2,4-epibrassinolide^5^. Alternatively, pectin-derived oligosaccharides^6^, kaolin foliar fertilizer^7^, and cyanocobalamin^8^ can be used to improve grape berry color development.

In recent years, biostimulant have been widely used to horticultural crops as they have plant growth-promoting effects. Biostimulant is “a formulated product of biological origin that improves plant productivity as a consequence of the novel, or emergent properties of the complex of constituents, and not as a sole consequence of the presence of known essential plant nutrients, plant growth regulators, or plant protective compounds”^9^. The biological basis of biostimulants is compounds from the diverse sources, which include bacteria, fungi, seaweeds, higher plants, animals and humate-containing raw materials^9,10^. Several studies have investigated the effects of biostimulant on grape growth^11–13^. These results found that biostimulant enhanced the foliar ion uptake, improved stress resistance, and increased the berries pigment contents by possibly stimulating the phenylalanine pathway. However, compared with the demonstrated efficiency of phytohormones (for example, ABA or its isomer) for anthocyanin accumulation in the skins of several grape cultivars, such as ‘Benitaka’, ‘Crimson Seedless’, and ‘Isabel’^14–16^, the accurate effects of biostimulant on both the expression of structural and regulatory genes in the flavonoids pathway and the color development of berries have been little investigated.

The present study consisted of a biochemical analysis, measurement of enzyme activities, and determination of changes in gene transcription levels to assess the influence of SUNRED product (one type biostimulant) application on grape berries color development. Additionally, the effects of the SUNRED were compared with those of S-ABA, which was applied widely in horticultural crop for improving fruit quality. These analyses will provide insights into the effects of exogenous SUNRED product on anthocyanin biosynthesis and accumulation in the skin of table grapes and elucidate how this treatment regulates anthocyanin biosynthesis. The data obtained here will aid the practical application of biostimulant for grape quality improvements in the field.

## Materials and Methods

### Field conditions

The experiment was carried out in a commercial vineyard in Pengshan county(N30°14′41″, E103°51′39″), Meishan, China, where is subtropical warm-moist climate with average annual rainfall of 983.4 mm, annual average temperature of 17.1 °C, and relative humidity of 82%. Seven-year-old ‘Red globe’ grapevines (*Vitis vinifera* L.) were used as materials. The grapevines were spaced at 2 × 3 m and cultivated under rain-shelter. The experiment was performed in the 2015/2016 production cycle.

### Experimental design

A randomized block design was carried out with three blocks and three treatments, and each treatment in the block consisted of five individual vines. The different treatments were provided by varying the concentration of the S-ABA isomer (10·l^−1^ active ingredient of abscisic acid in the obtained concentrate, Longmangfusheng Sciences Co., China) and the SUNRED (Biolchim Co., Italy). SUNRED is a proprietary mixture containing 26.6 g·l^−1^ of organic N, 13.3 g·l^−1^ of mineral N, 93.1 g·l^−1^ of K_2_O, and 186.2 g·l^−1^ of organic C. These components derive from inorganic fertilizers (potassium salts, urea) and liquid plant extracts rich in oxylipins, phenylalanine, methionine, and monosaccharides. The raw materials of SUNRED product are water, beetroot, potassium, urea, seaweed extract, glucose, amino acids, sodium hydroxide, citric acid. (https://www.biolchim.it/en/products/SUNRED/). The descriptive literature was used to select the S-ABA and SUNRED concentrations used in this study. The S-ABA concentrations were obtained by diluting the stock solution 100-fold (A100), 200-fold (A200), and 300-fold (A300). Similarly, the SUNRED stock solution was diluted 600-fold (S600), 800-fold (S800), and 1000-fold (S1000). Pure water treatment was used as the control (Mock treatment). Tween 80 was added as a wetting agent at 1 ml·l^−1^ in all treatments and control.

At the start of *véraison* in local region (about July 15, approximately 55 days after anthesis, DAA), S-ABA and SUNRED were applied directly to the leaves and berries with a handheld sprayer until runoff. The treatment was repeated 5 days later. Berries were collected at 60, 65, 70, 80, 85, and 90 DAA. The skin and pulp of the berries were separated and immediately frozen in liquid nitrogen, subsquently stored at −70 °C until use.

### Harvest characteristics evaluation

Freshly picked bunches of berries were weighed using an analytical balance with a precision of 0.1 g. Juice characteristics were determined from a sample of 50 berries per plot: the soluble solids content (SSC) was measured by a pocket-refractometer, PAL-1 (ATAGO, Japan). Titratable acidity (TA) was determined by titration with 0.1N NaOH, using 5 ml of diluted juice and expressed as g tartaric acid 100 mL^−1^ juice. The color of the berries was evaluated in samples of 50 berries per plot, employing a Minolta colorimeter CR-10. The values of L^*^(luminosity), a^*^, and b^*^ were obtained by direct detection. The values of C* (chroma) and h° (hue angle) were calculated using a* and b*, C* = [a*2 + b*2]^1/2^, and h° = arc tangent (b*/a*).

The color index of red grapes (CIRG) was determined using the formula: CIRG = [(180 − h°)/(L* + C*)]. The net photosynthetic rate (*P*n) was evaluated from fully expanded leaves using a combined open gas-exchange system (*LI-6400-40, LI-COR*, *Inc*., Lincoln, NE, USA). Total chlorophyll was extracted with 80% acetone, and the concentration of chlorophyll a and b were determined at 663 nm, 647 nm, and 470 nm using spectrophotometer (Evolution 300, Thermo Fisher Scientific, USA), the sum of chlorophyll a and b is total chlorophyll, according to the method of Lichtenhaler and Wellburn^17^. Total anthocyanin content of the samples was determined at 520 nm, 700 nm using spectrophotometer (Evolution 300, Thermo Fisher Scientific, USA), according to the pH-differential method described by Orak^18^.

### RNA extraction and real-time qRT-PCR

Total RNAs were extracted from grape skins following the procedure described in Gasic *et al*.^19^. The quality of the RNAs was checked by agarose gel electrophoresis and quantified using a spectrophotometer (NanoDrop 2000, Thermo Fisher Scientific Inc.). First-strand complementary DNA (cDNA) was reverse-transcribed from 200 ng of total RNA using the PrimeScript™ RT Reagent Kit with gDNA Eraser (Perfect Real Time) (Takara). Real-time PCR was performed in triplicate on an iQ5™ Real-Time PCR detection system (Bio-Rad). Relative levels of RNA were estimated by the 2^−ΔΔCT^ method^20^. Actin (*β-actin*) and Ubiquitin conjugating factor (UbiCF) were used as reference genes for the relative quantification of PCR products (Table 1). Gene specific primer pairs used for each target or reference gene are listed on Table 1.

**Table 1:** Primers used in this study.

Gene	Sequence (5′ → 3′)	Accession number of reference genes deposited in NCBI
*PAL*	F:CCACTTCACATAGGAGAA R:ATATCCCAGCATTCAAGA	XM_002268696.3
*CHS*	F:CTTGGGACTGGGAGATTC R:AACGGTAGATATGCTTCCA	AB015872.1
*CHI*	F:TTGTGTTGGTTCCTCTTGTTC R:GCAGACGAATCTCATTCAGTATAG	NM_001281104.1
*F3H*	F: CTTGGATCACCGTTCAACCT R: CAGGGTTTTGGAATGTTGCT	X75965
*F3*′*H*	F: AAAACCTACGGCCCTCTCAT R: AGGAGGCCTGTTGGAGAAAT	AB213603
*F3*′*5*′*H*	F: GAAGTTCGACTGGTTATTAACAAAGAT R: AGGAGGAGTGCTTTAATGTTGGTA	DQ298201
*DFR*	F:TTGTAATGGTCAATGTGCC R:CATGCAGAGACCACCTTG	X75964.1
*ANS*	F: AGGGAAGGGAAAACAAGTAG R: ACTCTTTGGGGATTGACTGG	X75966
*UFGT*	F:TGCTACCTAAGGCGACTG R:GCTTGGATTTGAGATCATTGG	DQ513314.1
*MYBA1*	F:TAGTCACCACTTCAAAAAGG R:GAATGTGTTTGGGGTTTATC	AB097923.1
*MYBA2*	F:CTGGAGAGATGCTTATCG R:TCAGCTTACTGGAAGTACTTA	AB097924.1
*UbiCF*	F: CTATATGCTCGCTGCTGACG R: AAGCCAGGCAGAGACAACTC	CF203457.1
*Actin*	F:CGTACAACTGGTTCGTATT R:TAGAACTCAGGCAACACTA	AY680701.1

Note: phenylalanine ammonia-lyase (PAL), chalcone synthase (CHS), chalcone isomerase (CHI), flavanone 3-hydroxylase (F3H), flavonoid 3′-hydroxylase (F3′H), flavonoid 3′5′-hydroxylase (F3′5′H), dihydroflavonol 4-reductase (DFR), anthocyanidin synthase (ANS), UDP glucose: flavonoid 3-o-glucosyl transferase (UFGT), myeloblastosis (MYB), actin (β-actin) and ubiquitin conjugating factor (UbiCF).

### Enzyme activity detection

The activity of phenylalanine ammonia-lyase (PAL), chalcone isomerase (CHI), flavonoid 3-o-glucosyl transferase (UFGT), and dihydroflavonol 4-reductase (DFR) were measured according to the methods of Liu *et al*.^5^, Lister *et al*.^21^ and Stafford^22^.

### Data analysis

All statistical analyses were performed with SPSS 22.0. The effects were tested using one-way analysis of variance (ANOVA). The significance of the differences between the mean values of each treatment was determined according to Duncan’s multiple range test at p < 0.05. The results presented as mean ± standard error (SE). Figures were illustrated using Excel 2010.

## Results and Discussion

### Physicochemical aspects of photosynthetic rate (*P*n), total chlorophyll content, fruit weight, soluble solids, and titratable acid

There were significant differences among treatments for *P*n, total chlorophyll content of leaves, and weight of the berries (Table 2). For all of these endpoints, the largest values were obtained at full maturity stage after treatment with A100 or S1000.

**Table 2:** The net photosynthetic rate (*P*n) and total chlorophyll content of fresh leaves and weight, soluble solids, titrable acidity of berries.

Treatment	*P*n (μmol·m^−2^·s^−1^)	Total chlorophyll content (mg/g)	Berry weight (g)	Soluble solids °Brix (%)	Titratable acidity (%)	Solublesolids/Titatable acidity
A300	6.79 ± 0.49cd	20.90 ± 0.23b	13.45 ± 0.85d	15.97 ± 0.63c	0.50 ± 0.20a	31.94 ± 0.21b
A200	8.27 ± 0.63bc	21.02 ± 0.14b	14.18 ± 1.01c	15.33 ± 0.41e	0.45 ± 0.08a	34.07 ± 0.27b
A100	9.20 ± 0.09ab	23.34 ± 0.09b	14.82 ± 0.95b	17.65 ± 0.33b	0.40 ± 0.10a	44.13 ± 0.24a
S1000	10.50 ± 1.01a	30.04 ± 0.10a	16.30 ± 0.67a	18.00 ± 0.11a	0.45 ± 0.19a	40.00 ± 0.13a
S800	10.32 ± 0.59a	22.48 ± 0.28b	14.10 ± 0.79c	16.72 ± 0.47b	0.52 ± 0.33a	32.15 ± 0.41b
S600	10.14 ± 0.99a	21.81 ± 0.28b	14.11 ± 0.58c	15.60 ± 0.28d	0.57 ± 0.16a	32.15 ± 0.41b
Mock	6.52 ± 0.53d	18.62 ± 0.12c	13.26 ± 0.92d	15.25 ± 0.19e	0.45 ± 0.12a	33.89 ± 0.14b

Different letters indicate significant differences according to Duncan’s multiple range test (P < 0.05).

Significant differences were also found between treatments for content of soluble solids, titratable acidity, and relative soluble solids/acidity of the berries (Table 2). In all treatments, the highest SSC values were observed after application of A100 (17.65 °Brix) or S1000 (18.0 °Brix); these values differed significantly from the control (15.25 °Brix). The A100 S-ABA and S1000 SUNRED concentrations also gave smaller TA for the grape must, but there were no significant differences among different treatments or the control. Overall, our data indicated that treatment with S-ABA or SUNRED resulted in fruit of better quality compared with the control, and that this effect was particularly pronounced after the A100 and S1000 treatments.

Fruit ripening involves the well-orchestrated coordination of regulatory steps, such as accumulation of anthocyanins and sugars, degradation of chlorophyll and organic acids, cell wall softening, and synthesis of volatiles^23^. It has been shown that the application of exogenous ABA enhances the accumulation of several metabolites and degradation of other metabolites involved in fruit ripening, thereby accelerating this process^24,25^; therefore, ABA is regarded as a positive regulator of fruit ripening. The application of S-ABA to ‘Rubi’^2^ and ‘Isabel’^26^ grapevines showed significant differences in the fresh mass of berries and bunches, soluble solids, titratable acidity, and ratio of soluble solids/acidity. SUNRED application on ‘Cabernet Sauvignon’ and ‘Prosecco’ grape increased sugar accumulation in musts of both white and red wines without altering quality parameters^11^. The results of Yuan *et al*.^12,13^ indicated that application of SUNRED on berries can improve the quality of berries, such as soluble solids and total acids. In this study, our data also indicated that *P*n, total chlorophyll content, fruit weight, soluble solids, and titratable acid increased in ABA and SUNRED treated groups when berries had ripened fully, compared with control group. This may be the reason why the treated plants yielded a greater fruit mass and better fruit quality.

### Berry color characteristics

**Figure 1:**
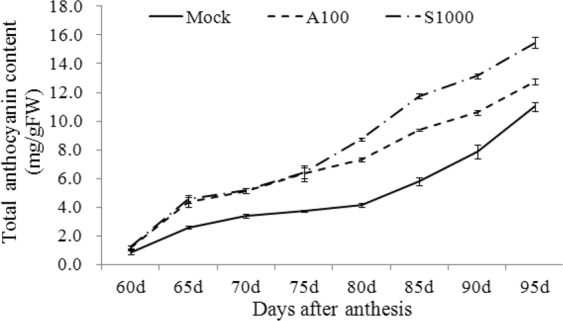
Total anthocyanin contents of berry skins after different treatments during berry development. The error bars represent Standard error (SE) (n = 3).

The anthocyanin contents of berries from the A100 and S1000 treatment groups, which had the best berry quality among all treatments, were significantly increased (Fig. 1). In this study, *véraison* had started by 55–60 DAA; therefore, anthocyanin concentrations were recorded from 60 DAA to 95 DAA (the whole ripening stage). Figure 1 shows the changes in anthocyanin accumulation in grape skins from A100 and S1000 treated fruit. The patterns of anthocyanin accumulation were similar in all treatments, with reaching a peak at 95 DAA. Compared with the control, there was an obvious increase in the anthocyanin contents in skins from S-ABA or SUNRED treated plants. A similar result for total anthocyanins in berries and grape juice was reported after S-ABA application on ‘Flame Seedless’^27^, ‘Alachua, ‘Noble’^28^, ‘Isabel’^26^ and SUNRED to ‘Red Globe’^12,13^.

Significant differences were found in the colorimetric variables and characteristics such as luminosity (L*), saturation (C*), hue angle (h°), and color index (CIRG) (Table 3). For color luminosity (L*), slightly differences were observed. The A100 or S1000 treatments presented smaller values (28.39 and 27.7) than the control (29.69), indicating that the berries were darker in the A100 or S1000 treated groups than the control, which had green colored berries. Higher values a* values and lower b* values were detected after the A100 or S1000 treatments; therefore, the berries in which groups showed higher saturation values (6.42 and 6.62) and lower hue angle values (23.45 and 20.60) than the control group. These changes indicated that berries in the treated groups had more pure and amaranthine color than those of the control group. The color index of red grape (CIRG) is use to evaluate the effects of S-ABA or SUNRED on the color of the full ripe grapes. In this case, there was a significant difference in CIRG, confirming that the A100 and S1000 treatments resulted in larger values (4.51 and 4.69) than the control treatment (4.15). Similar results after treatments with ABA were reported for the ‘Rubi’ grapevine by Roberto *et al*.^14^ and Neto *et al*.^2^, as like this in ‘Red Globe’ grapevine^12^.

**Table 3:** The parameter of colour characteristic of grape berries.

Treatments	a*	b*	L*	C*	h°	CIRG
Mock	5.32	3.15	29.69	6.20	30.63	4.15c
A100	5.87	2.52	28.39	6.42	23.54	4.51b
S1000	6.20	2.24	27.70	6.62	20.60	4.69a

Note: Different letters indicate significant differences according to Duncan’s multiple range test (P < 0.05).

### Transcriptional regulation of genes involved in anthocyanin biosynthesis in response to treatments with exogenous S-ABA and SUNRED

**Figure 2:**
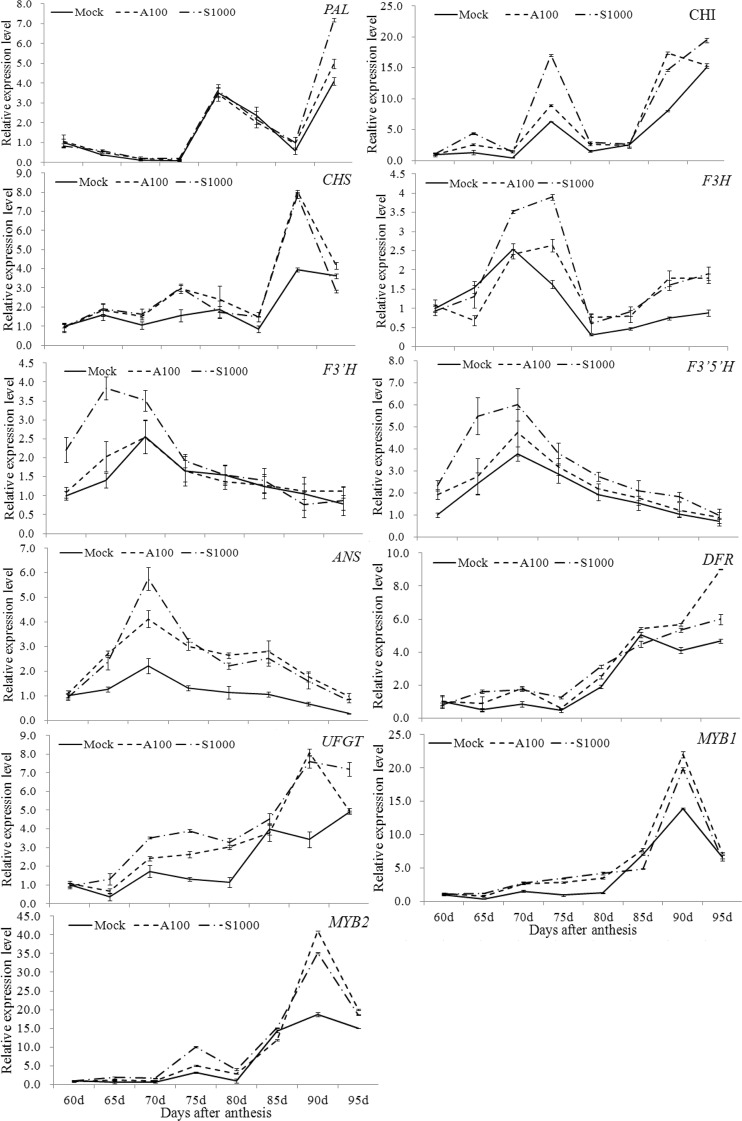
Effect of absicisic acid (ABA) and SUNRED on transcript levels of genes involved in anthocyanin biosynthesis pathways in the skin of berries. The error bars represent Standard error (SE) (n = 3).

The data described above indicate that exogenous S-ABA and SUNRED treatments significantly altered the anthocyanin contents of berry skins. To investigate the effect of exogenous S-ABA and SUNRED on the regulation of anthocyanin biosynthesis, the transcript levels of 11 genes involved in anthocyanin metabolism pathways were analyzed in the A100 and S1000 treatments (Fig. 2).

The qRT-PCR analyses showed that ABA or SUNRED treatments altered the relative expression levels of genes in the anthocyanin biosynthesis pathway. Compared with the control, expression of all tested genes was up-regulated by A100 or S1000 treatments, either at particular stages or throughout the fruit ripening process. For example, the transcript levels of phenylalanine ammonia-lyase (*PAL*) were only higher at 90–95 DAA after A100 and S1000 treatments compared with the control, while the transcript levels of chalcone synthase (*CHS*) and chalcone isomerase (*CHI*) were higher in the treatment groups than the control during almost all the fruit ripening process.

The A100 and S1000 treatments had similar effects on the expression patterns of almost all the tested genes. Two peaks (mid- and late-period) of expression were seen for *PAL* and *CHI* during the process of fruit ripening. Expression of *CHS*, dihydroflavonol 4-reductase (*DFR*), UDP glucose: flavonoid 3-o-glucosyl transferase (*UFGT*), *MYB1* and *MYB2* remained low at the first three stages of ripening, but were elevated at the final ripening stage in both control and S-ABA/SUNRED treated fruits, suggesting that these genes function mainly in the post-maturation stage. The gene expression levels of flavanone 3-hydroxylase (*F3H*), flavonoid 3′-hydroxylase (*F3*′*H*), flavonoid 3′5′-hydroxylase (*F3*′*5*′*H*) and anthocyanidin synthase (*ANS*) were upregulated during the early-to-mid period of fruit ripening, but showed a consistent decrease until the fruit fully ripened; the expression levels at 90–95 DAA were higher than those at 60–65 DAA.

Exogenous hormone or foliage fertilizers are a widely-used and effective method to improve the color of berries; brassinosteroids have been applied to ‘Cabernet Sauvignon’ grapevines by Luan *et al*.^4^, pectin-derived oligosaccharides to ‘Cabernet Sauvignon’ by Villegas *et al*.^6^, and cyanocobalamin to ‘Crimson seedless’ grapevines by Lo’ay^8^. These studies showed that the plant hormone or foliage fertilizer plays an important role in the color of mature grapes through increasing the level of transcription of several color-related genes, such as *PAL*, *DFR*, *CHI*, *F3H*, *GST*, *CHS*, and *UFGT*. In the present study, S-ABA and SUNRED treatments also appeared to increase the expression of genes involved in anthocyanin biosynthesis pathway. Our results also indicated that SUNRED had a greater effect on berry color than S-ABA, as gene expression levels were higher in the former treatment group. Taken together, these results indicated that exogenous ABA and SUNRED could influence gene transcription levels to induce anthocyanin accumulation.

### Effects of exogenous ABA and SUNRED treatments on enzymes involved in anthocyanin accumulation during fruit ripening

**Figure 3:**
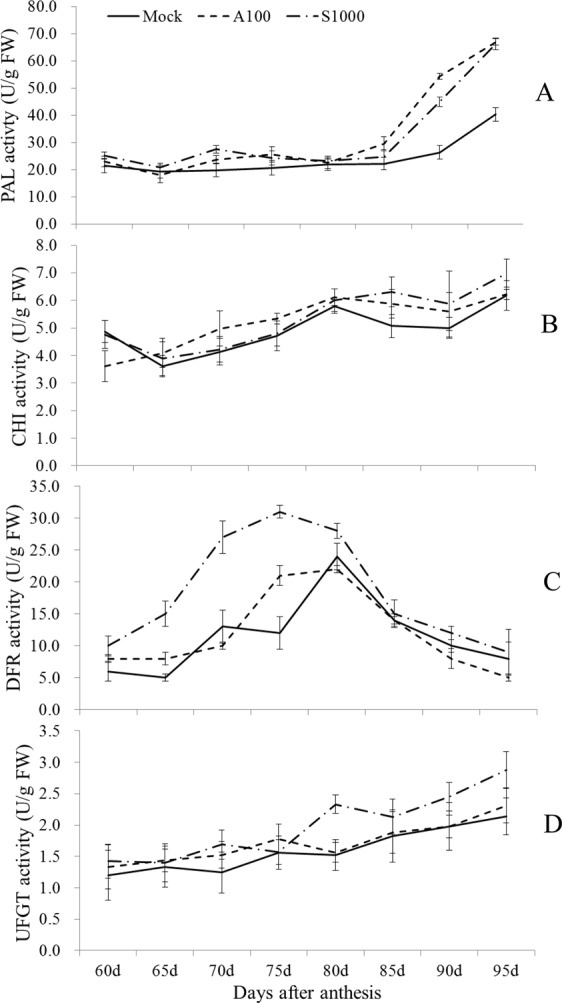
Effect of absicisic acid (ABA) and SUNRED on activity of the enzymes involved in anthocyanin biosynthesis in the skin of berries. The error bars represent Standard error (SE) (n = 3).

The effects of A100 and S1000 on activities of PAL, CHI, DFR, and UFGT during 60–95 DAA are shown in Fig. 3(A–D). PAL activity was significantly stimulated by A100 and S1000 treatments compared to the control (Fig. 3A). There was a clear increase in PAL activity from 85 DAA to 95 DAA in all treatment groups. S-ABA and SUNRED application caused a further increase at later period in PAL activity compared to the control. An increase from 60 DAA to 75 DAA following a drop of DFR activity was found over the artificial exogenous spraying trial (Fig. 3C). Treatment with S1000 induced a significantly higher DFR activity than in the control or A100 treatments over the whole fruit ripening process. CHI and UFGT activities showed continuous increases in this experiment (Fig. 3B,D). The A100 and S1000 treatments showed significantly higher CHI and UFGT activities than the control during the berry ripening period. Both CHI and UFGT activities were higher after the S1000 treatment than the A100 treatment at the later period of fruit ripening.

### Hypothetical pathway for the effect of SUNRED on regulation of anthocyanin biosynthesis

According to the Yakhin *et al*.^9^, du Jardin *et al*.^10^ and Caradonia *et al*.^29^, categories of plant protection products/compounds and fertilizers are very widely. Including two main parts, one is chemically characterized products, such as plant growth regulators, mineral fertilizers; another is non- or partly chemically characterized complex products derived from natural raw material, for example, bio- or organic fertilizers, biostimulants. A plant biostimulant is any substance or microorganism applied to plants with the aim to enhance nutrition efficiency, abiotic stress tolerance and/or crop quality traits, regardless of its nutrients elements. The biostimulant derives from natural raw material, including seaweed and higher plants extracts, animal substances, chitin and chitosan, complex organic mixtures, humates, trace elements, microorganisms and their metabolites. Therefore, from these concept and the introduction via Biolchim Co., we considered the SUNRED product as a kind of biostimulant, containing inorganic fertilizers (potassium salts, urea).

These components of SUNRED derive from inorganic fertilizers (potassium salts, urea) and liquid plant extracts rich in oxylipins, phenylalanine, methionine, and monosaccharides. Seaweed extracts is important source for SUNRED product. Several studies have shown that foliar application of seaweed extracts can increase the photosynthetic capacity and chlorophyll content of leaves^30,31^. The increased efficiency of photosynthesis results in the production of more photosynthates (monosaccharides) that can react with anthocyanidins to form anthocyanins. Seaweed extracts contain phytohormones such as indole acetic acid, cytokinins, gibberellic acid, polyamines, and abscisic acid^31^. Many genes involved in plant growth and development are regulated by phytohormones, which can discern *cis-motifs* in the promoter sequences of the genes^32^. These results indicate that SUNRED product can increase the expression of genes involved in anthocyanin biosynthesis possibly through stimulation of promoter activities of these genes.

Moreover, the high levels of oxylipins, phenylalanine, and monosaccharides in SUNRED product may be another important source for formation of anthocyanins. Phenylalanine serves as precursors to form anthocyanidins, then anthocyanidins are glycosilated by monosaccharides to form stable anthocyanins^33^. SUNRED product also supplies the oxylipins as enhancers for a lot of cyclopentanonic compounds involved in several ripening-related processes, such as chlorophyll degradation, anthocyanins and polyphenol accumulation^34^.

Potassium is one of the most abundant cations in grapes^35^. Potassium is essential for growth, enzyme activation, photosynthesis, osmotic regulation of grape^36^, also for yield and quality of berries^37,38^. The commercial SUNRED product contains a range of inorganic potassium, which plays key roles in catalyzing enzyme activities involved in anthocyanins biosynthesis of berries. In this light, it is a reasonable expectation that SUNRED product can enhance anthocyanins content in berries skin, as found in this study.

In conclusion, our study presents novel information on the modifications of total anthocyanin content in berry skins ‘Red Globe’ grape in response to exogenous SUNRED product (biostimulant) application under field conditions. The increase in anthocyanin content was associated with increased levels of expression of genes in the anthocyanin biosynthesis pathway and of their enzyme activities. We also show that a single SUNRED application at the onset of *véraison* had a better effect on berry quality than S-ABA application. Hence, the preharvest application of biostimulant, for example SUNRED product, at the star of *véraison* is an effective method for improving anthocyanin pigment in grapes.
